# Is there a sicker sex? Dose relationships modify male–female differences in infection prevalence

**DOI:** 10.1098/rspb.2023.2575

**Published:** 2024-01-10

**Authors:** Nathan J. Butterworth, Lindsey Heffernan, Matthew D. Hall

**Affiliations:** School of Biological Sciences, Monash University, Wellington Road, Clayton, Victoria 3800, Australia

**Keywords:** sex differences, sexual dimorphism, host–pathogen interactions, susceptibility, disease ecology, epidemiology

## Abstract

Throughout the animal kingdom, there are striking differences in the propensity of one sex or the other to become infected. However, precisely when we should expect males or females to be the sicker sex remains unclear. A major barrier to answering this question is that very few studies have considered how the susceptibility of males and females changes across the full range of pathogen doses encountered in nature. Without quantifying this ‘dose–susceptibility’ relationship, we have likely underestimated the scope for sex differences to arise. Here, we use the *Daphnia magnia*–*Pasteuria ramosa* system to reveal that sex differences in susceptibility are entirely dose-dependent, with pathogens having a higher probability of successfully establishing an infection in mature males at low doses, but mature females at high doses. The scope for male–female differences to emerge is therefore much greater than previously appreciated—extending to sex differences in the upper limits to infection success, per-propagule infectivity risks and density-dependent pathogen behaviour. Applying this expanded scope across the animal kingdom will help us understand when and why a sicker sex emerges, and the implications for diseases in nature—where sex ratios, age structure and pathogen densities vary drastically.

## 1. Introduction

Males and females differ in their genetics, physiology and behaviour—all of which can alter the likelihood and severity of infection [1–6] and the spread of pathogens [7–12]. Many attempts to define whether males or females are more likely to be damaged by a pathogen and become the ‘sicker sex’ have centred on host susceptibility (i.e. the likelihood of infection at exposure), as this is the stage when pathogen encounter and sex differences in immunity first collide [13–15]. While in some taxa males appear to be more susceptible to infection [1,16–20], there are many examples where it is females instead [12,21–24]. Thus, while sex differences in susceptibility are widespread, attempts to identify the consistently ‘sicker' sex have proven challenging [3].

**Figure 1.**
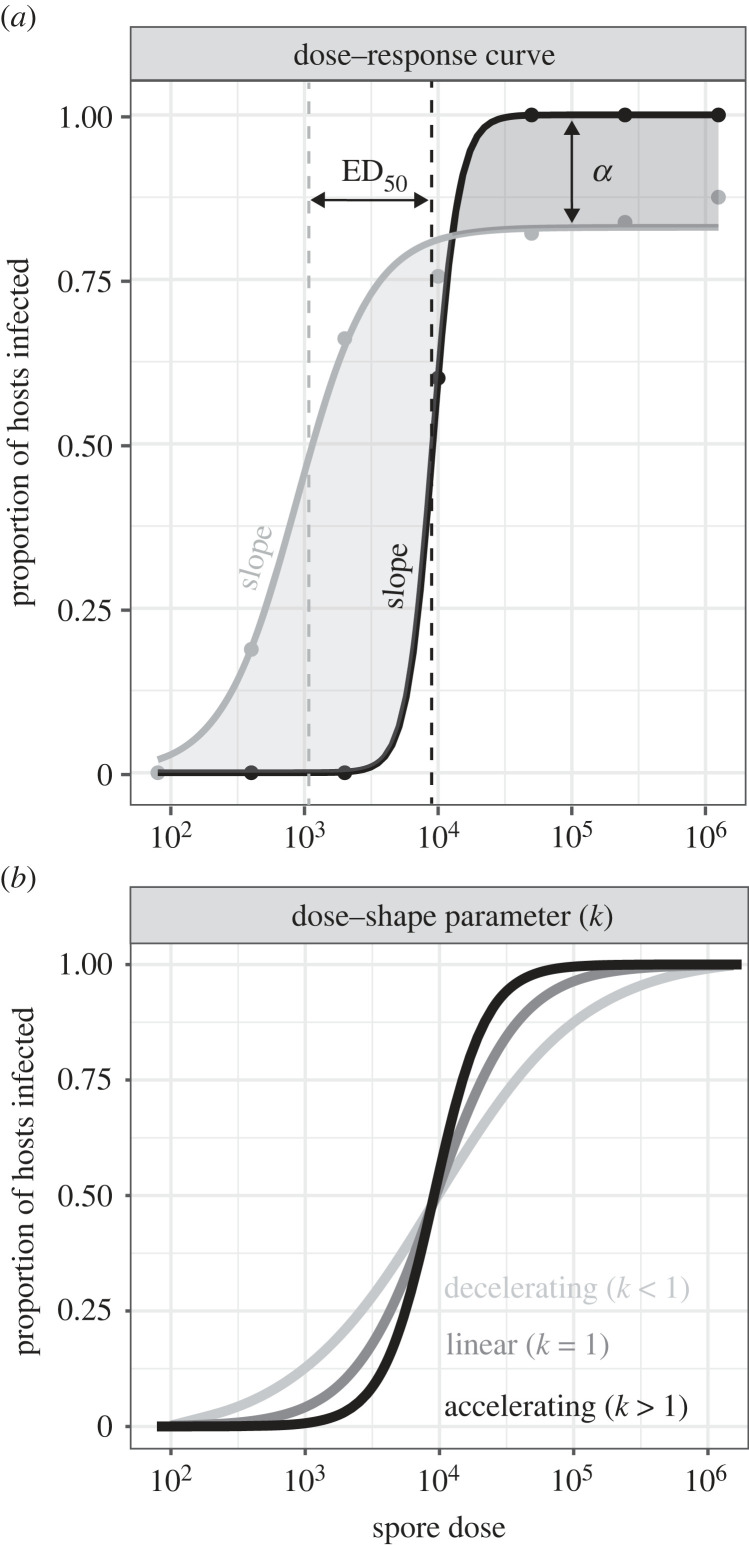
Quantifying dose–susceptibility relationships gives rise to a range of parameters that can lead to biologically meaningful interpretations. (*a*) Parameters that can vary between dose–response curves and are quantifiable include: α, the reduction in the upper boundary limit to infection success (proportion of hosts that are not infected); ED_50_, the effective dose at which 50% of hosts are infected (equivalent to the median susceptibility); and the slope of the curve (related to the variance in susceptibility and the establishment threshold of the pathogen). (*b*) A representation of how changes in the dose-shape parameter (*k*, estimated via the parasite synergism/antagonism model of [33]) alter the dose–response relationship when other parameters are constrained. These include a proportional relationship (*k* = 1; equivalent to mass action), an accelerating relationship (*k* > 1; pathogen synergism), and a decelerating relationship (*k* < 1; pathogen antagonism). All these components may differ between sexes but have yet to be formally analysed.

In understanding when and why a sicker sex emerges, one major factor underpinning the likelihood of infection has been overlooked—how male and female susceptibility changes with pathogen dose [25–31]. This relationship between pathogen dose and susceptibility has been commonly explored by exposing a standardized number of hosts (typically individuals) to different numbers of infectious units of a pathogen [25,30,32,33]. The value of this ‘dose–response' data [34,35] is that it provides insight into aspects of susceptibility that are completely invisible in single-dose experiments. For example, by measuring infection success across multiple doses, we can quantify (i) the lowest dose that generates successful infections, (ii) the rate at which infection rates change with pathogen doses (i.e. the dose–response slope), (iii) the pathogen density required to infect 50% of hosts (ED_50_), and (iv) any upper limits to infection success for a pathogen (figure 1*a*).

Dose–response studies also provide insights into host–pathogen interactions that only emerge at specific pathogen densities. One way to achieve this is by comparing the observed dose–susceptibility relationship with a null model, such as the ‘mass-action’ model of infection [33,36]. Under the mass-action model, the likelihood of infection is directly proportional to the number of infectious units encountered by the host, as determined by a constant rate of infectivity per pathogen propagule (i.e. the transmission coefficient). Deviations from mass-action (see [27,30,37]) suggest that other biological processes may be influencing infection rates at any given dose. For instance, if the infected fraction never reaches unity, it may suggest an upper limit to infection success or an underlying cohort of resistant hosts ([27,33]; figure 1*a*). Alternatively, as dose increases, synergistic (accelerating) or antagonistic (decelerating) changes in infection can emerge [30,33,37]. For a pathogen, an increase in dose can make it either easier to infect hosts (synergistic), for example as immune defences are overwhelmed, or harder to infect hosts (antagonistic), for example as competition between pathogens begins to interfere [32,38]. This deviation from expected infection rates can be quantified by the additional ‘dose-shape' (*k*) parameter of the parasite synergism/antagonism model of infection (figure 1*b*; [33]).

As it stands however, none of these metrics has been quantified between the sexes. Most inferences on sex differences have been drawn from studies of wild populations (e.g. [20,39,40]) where underlying pathogen dose can only be approximated [41], or from laboratory studies where sex-specific infection rates are often inferred from limited doses (e.g. [16,17,19,23,42]). In fact, in a recent survey of pathogen dose–response studies [30], fewer than 5% of studies included both males and females, and none formally analysed sex differences in the dose–susceptibility relationship. Quantifying dose–susceptibility relationships for males and females thus offers new opportunities to expand the scope for sex differences to arise beyond simple infection rates. In particular, the dose-shape parameter (*k*) provides an entirely overlooked dimension where meaningful sex differences might arise, allowing sex biases in infection rates to become uncoupled from dose, such that differences between the sexes that are apparent at low doses reverse, or even become obscured, at high doses (see shading in figure 1).

Our current view of susceptibility differences between the sexes is therefore based only on a narrow window of the full dose–susceptibility relationship. To demonstrate how our understanding of sex biases in infection rates can be expanded, here we conduct a dose–response study using the facultatively parthenogenetic crustacean *Daphnia magna* and its bacterial pathogen *Pasteuria ramosa.* Previous work with this system has pioneered the use of dose–susceptibility analyses [27,32,33,37], through which it is well established that the likelihood of infection is tightly linked to pathogen dose and host physiology [27]. Males have been shown to be more resistant to infection than females at a limited range of doses [23], and the extent of resistance changes with age [23,42]. We therefore expect juveniles of both sexes to share similar patterns of susceptibility, but for sexually mature males to be broadly more resistant to infection than sexually mature females. However, whether this will hold true for a wider range of pathogen doses (i.e. the entire dose–response curve), and when we consider the multivariate view of sex differences in susceptibility offered by formal models of infection, remains unclear. By disentangling dose, sex and susceptibility, we will provide a formal framework for comparing sex differences in susceptibility within and between species.

## 2. Methods

### (a) Study organisms

*Daphnia magna* Straus is a freshwater crustacean that reproduces via facultative parthenogenesis and can produce genetically identical male and female clones [43]. The species is endemic to ponds throughout the Holarctic region [44,45]. *Pasteuria ramosa* Metchnikoff [46,47] is a natural Gram-positive bacterial pathogen of *D. magna*, which invades the host via attachment to the oesophagus and subsequently reproduces within the haemolymph of the infected *Daphnia*. Following infection, *P. ramosa* moves through a series of developmental processes and eventually fills the body with mature transmission spores, which are formed approximately 14–18 days after initial exposure [27,37,47]. Crucially, it is only these mature transmission spores that can generate new infections, as prior developmental stages are unable to infect new hosts [47]. In females, infection results in a severe reduction in fecundity and lifespan, and an increase in body size [48,49]. By contrast, males are naturally smaller, considered more resistant to infection, allow fewer spores to be produced, and suffer lower reductions in lifespan [23,42,50]. In both males and females, transmission occurs exclusively horizontally, after the release of spores from a dead host [10,51].

### (b) Production of experimental animals

This experiment used stocks of *D. magna* of the genotype HU-HO-2, originating from a pond in Kiskunság National Park, Hungary, and *P. ramosa* of the genotype C19, originating from an infected female collected from a pond in Gaarzerfeld, Germany [52]. To minimize variation in maternal effects, standardized female *D. magna* were raised individually for three generations in 70 ml jars filled with 50 ml of artificial *Daphnia* medium (ADaM) ([53]; modified in [54]) and maintained under standard conditions (20°C, 16L : 8D).

Genetically identical male and female *D. magna* were produced by exposing the third generation of standardized females to the crustacean juvenile hormone methyl farnesoate (300 μg l^−1^; product ID: S-0153, Echelon Biosciences, Salt Lake City, UT, USA). Following established protocols, adult females were transferred to 20 ml of the hormone solution after the release of their first clutch [50,55]. The second clutch produced after exposure to the hormone, composed of a mixture of male and female offspring, was then collected and immediately returned to normal artificial medium. This method of producing both male and female *Daphnia* has no detectable impact on either the lifespan or fecundity of control animals [55]. Male and female *Daphnia* offspring were identified by the presence/absence of the sexually dimorphic appendage used for clasping onto females [43].

### (c) Dose–response experimental design

To assess sex differences in susceptibility across a range of doses for both juvenile and mature individuals, males and females to be infected were housed individually in 70 ml jars, filled with 50 ml of artificial medium. Animals were fed daily throughout the entire experiment with increasing numbers of algal cells (*Scenedesmus* sp.)—1 million cells (at 1 day old), 2.5 million cells (at 2–5 days old), 3 million cells (at 5–6 days old) and 5 million cells (from day 7 onwards). Animals were transferred into 50 ml of fresh ADaM every 3 days throughout the entire experiment. Each treatment consisted of 50 individual replicates for a total of 1400 experimental animals (2 sexes × 2 ages × 7 doses × 50 replicates).

To infect the animals, *P. ramosa* spores were set up as follows: the highest spore dose of 1 250 000 spores was subsequently diluted seven times by a factor of five to produce the other doses, resulting in seven dose levels of 80, 400, 2000, 10 000, 50 000, 250 000 and 1 250 000 spores. At 1 day of age, the ‘juvenile' cohort of animals were transferred individually into jars containing 20 ml of ADaM and dosed with one of the seven *P. ramosa* spore doses. After 4 days, animals were moved to fresh jars filled with 50 ml of ADaM and then the water was changed as usual after. At 10 days of age, the ‘mature' cohort of animals were transferred individually into 20 ml of ADaM and dosed with one of seven *P. ramosa* spore doses. Again, after 4 days, animals were moved to fresh jars filled with 50 ml of ADaM and then the water was changed as usual after.

### (d) Quantifying host susceptibility

Spores of *P. ramosa* infect the haemolymph of *Daphnia* and successful infection induces brownish coloration in both males and females [43,49]. These phenotypic changes make it easy to identify infection status, which can be determined approximately 14–18 days after initial exposure [27,37,47]. Experimental animals were thus monitored throughout the experiment until death, or eventually euthanized at 30 days, at which point all individuals had their infection status recorded via their appearance (as per [37]). In any ambiguous cases (such as animals that died before 14 days post-exposure), animals were crushed, and infection determined via the presence of transmission spores detected by haemocytometer counts under a phase-contrast microscope.

### (e) Analysis of dose–response data

All analyses were conducted in R (v. 4.2.1; [56]) and visualized using the *ggplot2* package (v. 3.4.0; [57]). A total of 31 animals were removed owing to being lost during the experiment. The final dataset therefore contained 1369 individuals across all seven doses (see electronic supplementary material). To test how host age and sex contribute to the dose–response relationship, we first modelled the change in the proportion of hosts infected with increasing spore dose as a three-parameter nonlinear logistic model via the package *drc* (v. 3.0-1; [35]), following the equation:

2.1 $$Y=\;\frac{d}{\left(1+e^{-b(X-e)}\right)}$$

where *Y* is the proportion of hosts that become infected (weighted by the total number of exposed hosts) and *X* is the log_10_ of the spore dose. This model allows us to partition susceptibility into three components (as per figure 1*a*): b, the slope and rate at which infections increase with *X*; *d*, the maximum asymptote (which captures the upper boundary limit to infection success; i.e. the maximum proportion of infected hosts); and *e*, the midpoint between the lower infection limit of 0 and the upper limit of *d*, often known as ED_50_ (i.e. the value of *X* at which 50% of susceptible hosts are infected).

For each age, we fitted this model twice, once where the parameters were constrained to be the same for each sex, and then a second time but allowing the parameters to vary by sex. We then used an analysis of variance to determine if the sex-specific parameter estimates improved the fit of the constrained model, and thus provide evidence that dose–response relationships varied by sex. Similarly, to determine if a model that includes an upper boundary limit is a better fit to the data for the mature age class, we fitted an additional model, where *d*, the maximum asymptote was constrained to 1 for both of the sexes, and compared this model with the sex-specific model with all free parameters using an analysis of variance.

### (f) Analysis of the parasite synergism/antagonism model

To assess whether the model of environmental transmission differed substantially from mass-action, and whether any changes might depend on the sex of the host, we formally extended our simple dose–response approach using the parasite synergism/antagonism model developed by Regoes *et al*. [33], and used previously by Ben-Ami *et al*. [37]. The equation for this model is as follows:

2.2 $$f_{j}=(1-\alpha )(1-e^{-\beta (P_{j}^{k})t_{\exp}})$$

where the fraction of infected hosts at a given dose $\left(f_{j}\right)$ is determined by any reduction in the upper boundary limit to infection success (α, from eqn 3 in [27] and therein referred to as the ‘resistant fraction'), the per-propagule infectivity of the pathogen (β), the pathogen dose $\left(P_{j}\right)$, the shape of the dose–response curve (*k*) and the time of exposure $\left(t_{\exp}\right)$. For *k* = 1, an increase in pathogen dose leads to a proportional increase in rate of infection (i.e. mass-action). Alternatively, for *k* < 1, an increase in pathogen dose leads to a decelerating increase in the rate of infection (an antagonistic effect of pathogen propagules), while for *k* > 1, an increase in pathogen dose leads to an accelerating increase in the rate of infection (a synergistic effect of pathogen propagules) [33].

The parasite synergism/antagonism model was implemented in R using the *CmdStanR* (V. 0.5.3; [58]) implementation of the Stan modelling language [59] and fitted to the experimental data. We assumed uniform priors based on a plausible range of values (following [28] and [30]). Convergence of the chains was checked visually, and the 90% credible intervals for each parameter were estimated by sampling from the posterior distribution.

Importantly, the interpretation of this model and data depends on two key assumptions that arise from the exposure trials occurring over a span of time (as opposed to hosts being exposed to all spores instantaneously), which is a common design for this and related study systems (see also [27,33,37,60–65]). First, this approach assumes that spores do not decay over the 4 day exposure period, which is reasonable as spores of *P. ramosa* can survive several decades in the field ([66]; as per [27]). Second, it also assumes that the consumption of spores by the host does not drastically alter the environmental density of spores over the duration of the infection trial.

Regarding the latter assumption, *Daphnia* can filter up to 20 ml per day during feeding [67], meaning that spore density could be changing rapidly over the infection period (4 days). Assuming that spore density remains constant could lead to biased parameter estimates—in line with how estimation of the functional responses of predators can be biased by not accounting for the changes in prey populations over time [68]. Fortunately, these issues are reduced in the *D. magna*–*P. ramosa* system because during feeding only a small portion of spores are trapped and bound to the host epithelium [23,47] while the remaining non-activated portion of spores remain viable and pass through the host [69]. Thus, not all spores are removed during feeding—unlike prey depletion due to the feeding rates of predators. Nonetheless, by modifying our approach to explicitly include host feeding rates, we can also show that our estimated parameters *α*, *β* and *k* are robust to a wide range of biologically relevant feeding rates (see electronic supplementary material, S1), a result that was also shown in Clay *et al*. [30]. As such, we opt not to include such a spore consumption effect in our main approach, instead using the more general model in line with previous dose–susceptibility studies [27,33,37].

### (g) Quantifying the impact of host frailty

**Figure 2.**
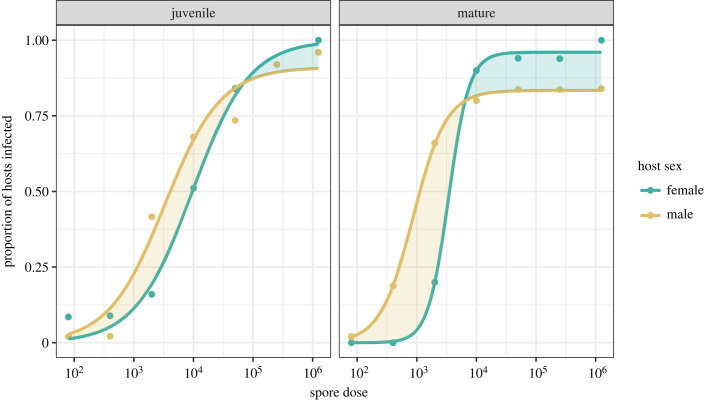
The nonlinear relationship between pathogen dose and infection success. Shown is the change in proportion of infected *Daphnia magna* (genotype: HU-HO-2) hosts in relation to the dose of the pathogen *Pasteuria ramosa* (genotype: C19), as modelled by a three-parameter logistic model with log_10_ spore dose as the explanatory variable. Juvenile individuals were exposed to the pathogen when 1 day old, mature individuals were exposed when 10 days old. Yellow shading represents a male bias in the probability of a pathogen establishing a successful infection. Green shading represents a female bias. For the same graphs with 95% confidence intervals plotted refer to electronic supplementary material, figure S3.

Lastly, to determine the contribution of host frailty to observed patterns of susceptibility, we also assessed the contribution of increasing spore dose to different aspects of host mortality. To test for sex differences in the change of mortality with increasing spore dose, we first modelled host mortality for all exposed hosts (as represented by the proportion of deaths prior to the 30 day experimental cut-off) using the three-parameter logistic model and modelling process as before (equation (2.1)). To explore the role of dose in driving these mortality differences between the sexes in more detail, we then partitioned the mortality data into hosts exposed to either low (80–2000 spores) or high (10 000–1 250 000 spores) levels of pathogen spores. We chose the cut-off between low and high doses based on where the plateau in infection success appears to begin (figure 2). Here, mortality (modelled as a binary outcome) and time until host death (modelled as a continuous outcome) were analysed as a generalized linear model (binomial error distribution, logit link function), and linear model, respectively. The significance of effects was then tested using analysis of variance (type III). Predicted means and standard errors were estimated using the *emmeans* package (V. 1.8.3; [70]).

## 3. Results

### (a) Sex biases in infection rates are dose-dependent and arise in mature animals only

To characterize how sex differences in the relationship between pathogen dose and infection rates might arise, we first estimated each dose–response curve using a three-parameter logistic model. For juvenile *Daphnia,* we found no clear statistical support for differences between males and females in their dose–response curves and underlying parameters (*F*_3,8_ = 2.201, *p* = 0.166). Thus, sex differences in susceptibility do not appear to arise in juvenile *Daphnia* at any dose (figure 2). For mature *Daphnia*, however, allowing parameters to vary by sex significantly improved the fit of the dose–response model compared with the model that constrained the curves to be equal for males and females (*F*_3,8_ = 120.190, *p* < 0.001), and a model that allows the upper boundary limit to vary by sex provided a better fit compared with the model that constrained the upper boundary limit to an equal value for both sexes (*F*_1,8_ = 72.254, *p* < 0.001). Overall, sex differences arose in the slope (steeper in females; females = 2.502 ± 0.63, males = 1.536 ± 0.30), upper boundary limit (higher in females; females = 0.960 ± 0.03, males = 0.842 ± 0.03) and ED_50_ (higher in females; females = 8.135 ± 0.16, male = 6.791 ± 0.17) parameters. As a result, mature males were broadly more susceptible to infection than mature females at low to medium doses, but at high doses the pathogen was unable to successfully establish transmission spores in a portion of mature males (figure 2).

### (b) Ontogenetic changes in environmental transmission occur predominantly for females

**Figure 3.**
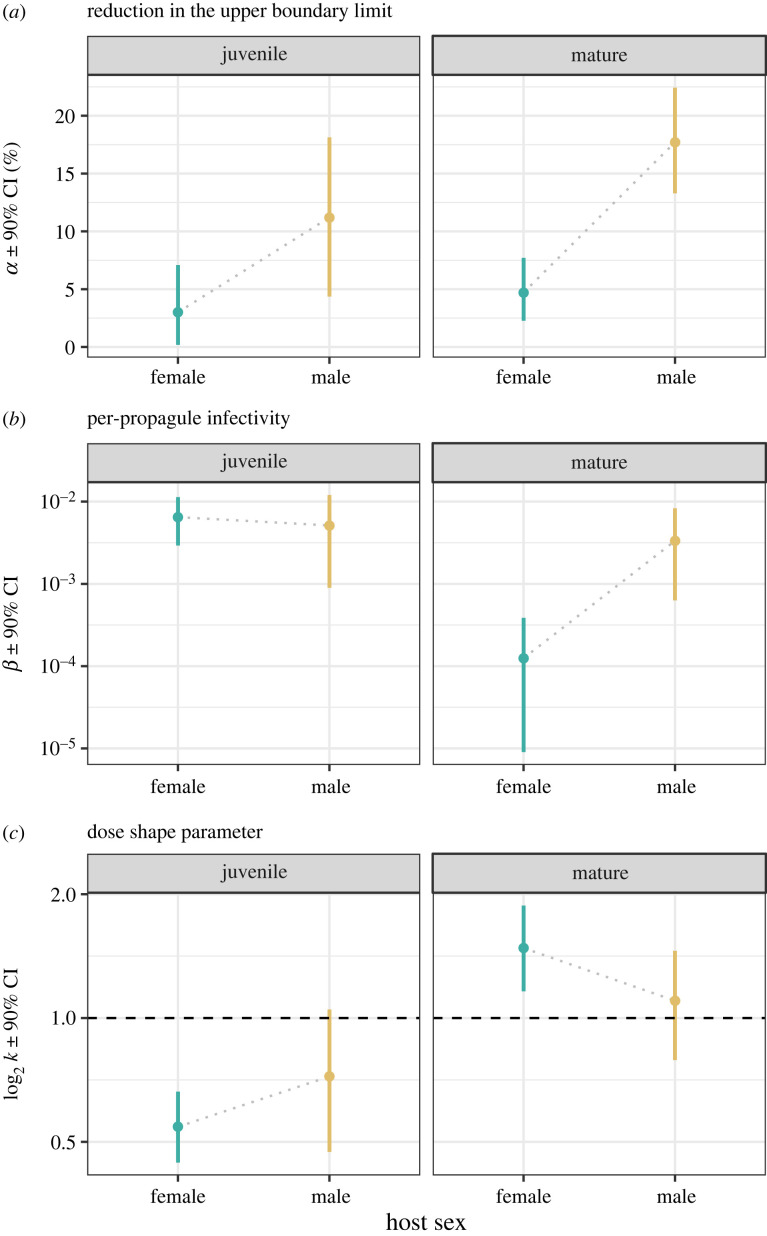
Parameter estimates derived from the parasite synergism/antagonism model of environmental transmission. Shown are the means and 90% credible intervals (CI) derived for each age and sex treatment combination. (*a*) α corresponds to the reduction in the upper boundary limit to infection success, (*b*) β corresponds to the per-propagule infectivity of the pathogen, and (*c*) *k* corresponds to the shape of the dose–response relationship and the potential for infection to deviate from mass-action (when k=1, dotted line).

To formally quantify these sex differences in terms of the different models of environmental transmission, we fitted the flexible parasite synergism/antagonism model of infection (equation (2.2); [33]) to our dose–response data. Beginning with the reduction in the upper boundary limit to infection success (as captured by α, figure 3*a*), males trended towards a higher α-value than females, with statistically clear differences emerging (non-overlapping credible intervals, CIs) at maturity. Pathogens infecting adult males thus have an upper limit to the probability of successfully establishing an infection. Likewise, for the per-propagule infectivity parameter (β, figure 3*b*), we again observe that clear differences between the sexes only occur at maturity, with females displaying over a 10-fold lower per-propagule susceptibility than males as an adult. By contrast, for males, the per-propagule susceptibility values did not change with ontogeny.

For the dose-shape parameter (*k,* figure 3*c*), we found that for females, infection followed an antagonistic relationship as juveniles (*k* ± 90% CI < 1) and a synergistic relationship as mature adults (*k* ± 90% CI > 1), indicating a substantial shift in the density-dependent processes shaping pathogen infectivity. For males, although their mean *k*-value generally increased across ontogeny, it was largely consistent with a mass-action model at both ages as the CI values indicate no statistically clear difference from a proportional dose–infectivity relationship (*k* = 1). Thus, in line with the previous analysis, the sexes did not differ as juveniles—but showed contrasting patterns through ontogeny, driven predominantly by greater changes in female susceptibility as reflected by both per-propagule infectivity (β) and a change in the dose-shape parameter (*k*), and to a lesser extent by a marginal change in the upper boundary limit (α) for males (but greater difference between the sexes as a result).

### (c) Mortality is dose-dependent and higher in males

**Figure 4.**
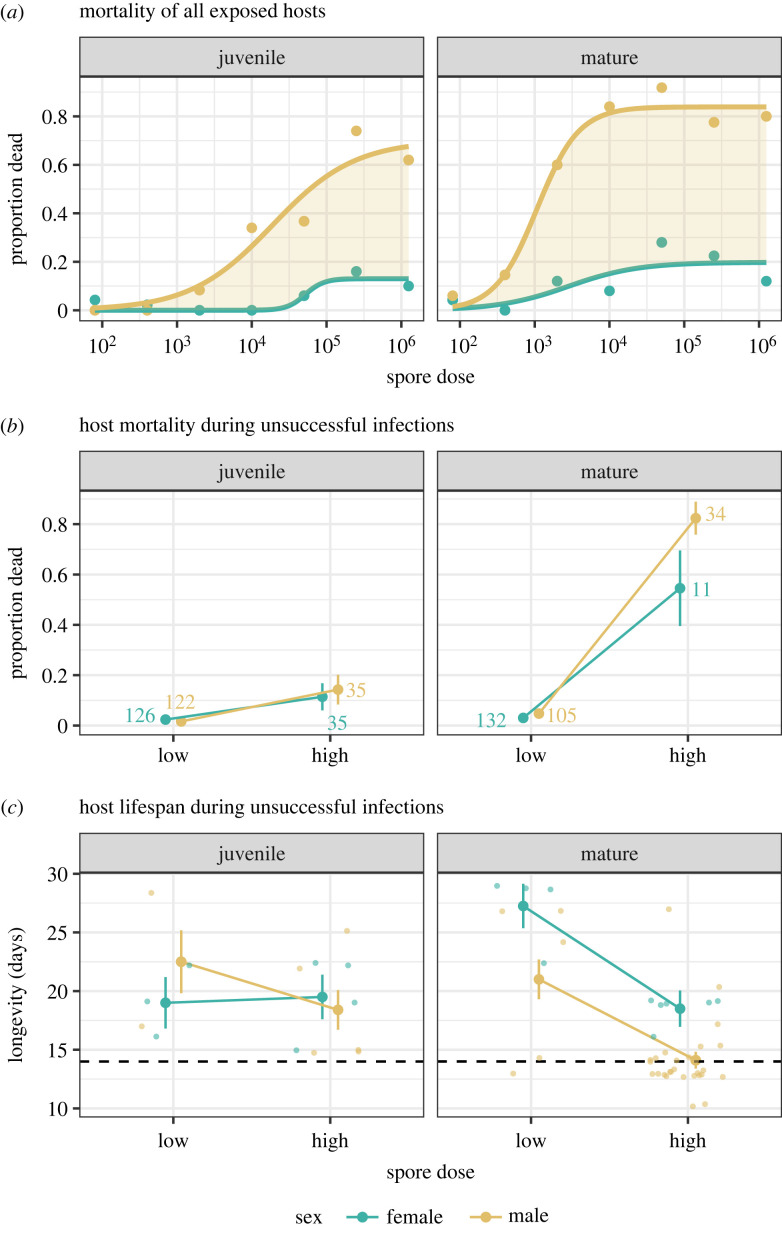
The effects of pathogen dose on the mortality and lifespan of male and female *Daphnia magna* at two different life stages (juvenile: infected at 1 day old; mature: infected at 10 days old). Shown in (*a*) is the proportion of hosts exposed to the pathogen that then died during the 30 day experiment, irrespective of their infection status. Curves were modelled with a three-parameter logistic function, with the yellow shading highlighting a male bias in mortality rates. For the same graphs with 95% confidence intervals plotted refer to electronic supplementary material, figure S4. In (*b*,*c*), the predicted means and standard errors are shown for (*b*) the proportion of host mortality during unsuccessful infections at low- and high-dose treatments, where the numbers next to means represent the total number of hosts from which the proportion was calculated, and (*c*) the post-exposure lifespan of hosts during unsuccessful infections and that died before the 30 day experimental cut-off. The small circles represent individual lifespan. Low doses constitute the 80, 400 and 2000 spore treatments. High doses represent the 10 000, 50 000, 250 000 and 1 250 000 spore treatments. The black dashed line represents 14 days, before which infection is not visually apparent in hosts and the production of mature transmission spores is not guaranteed [27,37,47].

A clear finding of our results is that a pathogen is unable to successfully infect and produce mature transmission spores in a fraction of males (the α fraction), even at very high exposure doses where infection in females would be completely assured. To determine what differences in male and female life history may be underlying these patterns, we considered how the mortality of males may be contributing to these results. Shown in figure 4*a* is the change in the proportion of exposed hosts that died during the 30 day experiment as partitioned by developmental age and sex. We find that the dose–mortality relationship diverged between the sexes for both juvenile (*F*_3,8_ = 26.302, *p* < 0.001) and mature *Daphnia* (*F*_3,8_ = 67.522, *p* < 0.001)*.* An increase in infection rates with dose, however, will naturally lead to dose-dependent increases in mortality, as infected individuals experience greater mortality due to pathogen virulence [42] and occur with more frequency at higher doses. Nonetheless, it is clear from figure 4*a* that males and females are not being affected equally, as the relationship between increasing dose and mortality (less than 30 days) is much stronger for males.

In principle, the α fraction could arise because either a host resisted infection to begin with, or the pathogen was prevented from producing mature transmission spores. To explore this, we considered the mortality and lifespan of hosts in which infections were not successfully established after being exposed to either low (fewer than 2000 spores) or high doses (more than 10 000 spores) of the pathogen (figure 4*b*,*c*). Our results show that variation in both frailty components depended on an interaction between dose and age (table 1). For unsuccessful infections in juveniles, there was a small, but significant, increase in mortality at high doses for each sex (figure 4*b* and table 1), but with no corresponding effect of pathogen dose or host sex on lifespan (figure 4*c* and table 1). By contrast, for mature hosts, both sexes were more likely to die during unsuccessful infections at the higher doses (dose effect *p* < 0.001) as well as experience a reduction in lifespan when unsuccessfully infected at high doses (dose effect, table 1), but males experienced a significantly greater reduction in lifespan than females overall (sex effect, table 1). These findings thus suggest that the α fraction males were likely to have been infected at high doses, but simply died before the pathogen was able to produce mature transmission spores—which require approximately 14 days to develop after pathogen exposure (dashed line in figure 4*c*; [47]).

**Table 1.** Result of the analysis of variance (type III) for the effects of dose (two levels: low or high), sex (two levels: male or female) and developmental age (two levels: juvenile or mature) on host mortality during unsuccessful infections (probability of death before experimental end) and host lifespan during unsuccessful infections (time until death after pathogen exposure) of uninfected hosts. Sloping numbers indicate significant values.

	host mortality during unsuccessful infections (figure 4*b*)			host lifespan during unsuccessful infections (figure 4*c*)		
full model	$\chi^{2}$	d.f.	*p-*value	*F*	d.f.	*p*-value
dose	59.24	1	*<0.001*	13.32	1.49	*<0.001*
sex	1.19	1	0.276	2.44	1.49	0.124
age	21.14	1	*<0.001*	0.08	1.49	0.783
dose ∗ sex	0.98	1	0.323	0.27	1.49	0.605
dose ∗ age	6.81	1	*0.009*	5.22	1.49	*0.027*
sex ∗ age	1.60	1	0.206	6.12	1.49	*0.017*
dose ∗ sex ∗ age	0.03	1	0.871	1.50	1.49	0.227
*juvenile*						
dose	12.06	1	*<0.001*	0.50	1.10	0.496
sex	0.01	1	0.915	0.22	1.10	0.648
dose ∗ sex	0.30	1	0.583	0.81	1.10	0.388
*mature*						
dose	62.63	1	*<0.001*	29.20	1.39	*<0.001*
sex	3.23	1	0.073	13.52	1.39	*<0.001*
dose ∗ sex	0.76	1	0.385	0.41	1.39	0.525

## 4. Discussion

Owing to the remarkable variability in the magnitude and direction of sex differences among species, attempts to generalize the more susceptible and ‘sicker' sex across the animal kingdom have proven challenging [3,71]. Many aspects of susceptibility, however, cannot be quantified in single-dose experiments [27,30]. Here, we explored how sex differences in susceptibility manifested across a wide range of pathogen doses, at two different ages, and formally quantified dose-dependent sex differences with a model of environmental transmission. Our original expectation (based on the prior results of [23]; but also see [42]) was that males of this species would be more resistant to infection as adults. Instead, we found that pathogen dose fundamentally altered the magnitude and direction of sex differences in susceptibility (figure 2). Based on the metric for which differences in susceptibility are commonly defined, the probability of becoming infected (e.g. [27,30]), pathogens had a higher probability of generating successful infections in males at low doses, whereas this pattern was reversed at high doses, with significantly higher numbers of successful infections in females. The expression of the ‘sicker sex' is thus entirely dose-dependent.

Our results reveal how the scope for sex differences to emerge in susceptibility is greater than previously expected and extends beyond simple measures of infection rates. Indeed, every metric of susceptibility that could be derived from a dose–response relationship was found to be sexually dimorphic in adult hosts. For some metrics, for example, males were more susceptible, as either they required a lower pathogen dose for 50% of the cohort to be infected (ED_50_, figure 2) or their per-propagule likelihood of infection was higher (β, figure 3). By contrast, in terms of an upper boundary limit to infection success (α, figures 2 and 3), infections established more readily in females, reaching 100% of hosts at high doses, whereas for a portion of exposed males, a successful infection was never established. Based on the dose-shape parameter (*k*), females also had a disproportionately higher likelihood of infection than expected by mass-action at higher doses, suggesting that females are more sensitive to the density-dependent nature of infection. Thus, no single component of susceptibility could define the ‘sicker' sex.

The emergence of sex biases in susceptibility for mature animals alone (figures 2 and 3) suggests that the degree of sexual dimorphism in susceptibility is linked to developmental processes (e.g. [15,17,72,73]). The most pronounced ontogenetic shifts occurred primarily in females for the per-propagule infectivity of the pathogen (*β*; which decreases for mature females), and how infectivity changes as pathogen density increases—as captured by the dose-shape parameter (*k*, figure 1*b*, [33]). Across both life stages, for example, males maintained a dose–susceptibility (*k*) relationship that could not be distinguished from the mass action model (i.e. *k* = 1), with the proportion of male hosts that become infected directly proportional to dose at all times. Females, however, exhibited a strong shift in *k* from an antagonistic relationship as juveniles (*k* = 0.54; higher spore doses lead to disproportionately lower infection rates than would be expected by mass-action) to a synergistic relationship as adults (*k* = 1.48; higher spore doses lead to disproportionately higher infection rates than would be expected by mass-action, figure 3*c*).

This sensitivity of the underlying model of environmental transmission to male and female differences, as captured by *k* and the synergistic–antagonistic model of transmission, presents an entirely new dimension for understanding the ‘sicker' sex. Previous studies using this very approach have shown how deviations from mass-action dose–response relationships can arise owing to maternal effects [27], or differences between species or genotype of host and pathogen [30,37]. Here, we demonstrate how, throughout development, one sex can be more sensitive to the density-dependent nature of infectivity (females in our case), leading to a threefold change in the nonlinear modifier *k*. This change, originating intrinsically from the sexes within a single species, is comparable in magnitude to what has been observed among entirely different host–pathogen systems (e.g. [30])—emphasizing the substantial effects that male–female differences can have on the dynamics of infection (see also [9–11,20]).

Underlying these observed sex-specific changes could be a range of behavioural and physiological differences that either modify the extent of heterogeneity in susceptibility among individual clones (as discussed in [33]) or shift the extent of density-dependent pathogen interactions [32,33] in one sex more than the other. For measures such as ED_50_ and per-spore infectivity (β), the lower susceptibility of adult females may reflect a greater investment in immunity than males, which is in line with how immunity and sex differences and roles are viewed from a sexual selection perspective [1,2,4,17,20,74,75]. Physiological differences such as feeding rates seem less likely to be contributing to these components of susceptibility, because female *Daphnia* have higher feeding rates than males in general [50], which should lead to a higher encounter rate with the pathogen (see [61]). So, in the case of per-propagule infectivity (β), females would be expected to show higher values than males, which they do not (figure 3*b*).

But why would females be the sex to exhibit substantial ontogenetic changes in their susceptibility profiles? While the exact mechanism underlying the greater change in susceptibility profiles for females and greater divergence between the sexes as adults is unknown, that females show greater phenotypic changes throughout ontogeny is consistent with sex-specific ‘ontogenetic trajectories' observed in many other animals, which probably reflect sex differences in life-history optimization. For example, males and females have been shown to differ in the ontogenetic trajectories of resource acquisition and allocation [76], the rate and duration of growth [77–79], and the timing of investment in different components of the immune response [80,81]. It is therefore not unexpected that one sex (females in this case) can show greater ontogenetic changes in the dynamics of infection. In fact, we have previously observed that female *D. magna* experience much greater shifts in pathogen virulence and spore loads in response to changes in resource availability [50], as well as greater changes in spore loads throughout ontogeny [10]. We now also provide evidence that female *D. magna* can have steeper ontogenetic trajectories in components of susceptibility (β and k).

It is difficult, however, to explain sex differences in a reduction in the upper limit to infection (α) by appealing to developmental changes in immunity or feeding rates alone—because at the highest doses any immune defences should be overwhelmed [27,32] and encounter rates with the pathogen maximized for both sexes. Indeed, if lower feeding rates mattered, we would also expect to see the highest values for α in juvenile males (which have the lowest feeding rates; [82,83]), but this is not the case (figure 3*a*). Contributing to α instead appear to be differences in the age-related frailty of mature males and females (figure 4*c*), with exposed males more likely to die before the pathogen can establish a successful infection and produce detectable spores (i.e. 14 days post- exposure or later; [47]). This is likely because at the time of exposure to the pathogen (10 days old), mature males are already one-third through their average lifespan (average lifespan 33 ± 1.9 days) versus only one-seventh for females (67 ± 2.0 days; [42,55]). Importantly, while these males are not functionally resistant, this is still an ecological dead end for the pathogen, and a net benefit to the host population, as these pathogens fail to generate the transmission spores needed for secondary infections [10,49]. Similar processes can be observed in bacterial systems where individual phage-infected bacteria self-sacrifice at an individual cost, but in turn reduce phage spread throughout the population [84].

Overall, by using *D. magna* and its bacterial pathogen as a test case, we have shown how a formal dose–response approach can greatly expand the scope with which sex biases in susceptibility can be considered. Dose-specific responses (e.g. ED_50_), per-propagule infectivity (β), changes to upper boundary limits (α) and even the underlying shape of transmission (k) are all different ways that a sicker sex can now be defined. These results help to explain why the more susceptible sex is so hard to characterize, even within a species, as the magnitude and direction of sex differences will depend entirely on pathogen dose, will vary with each component of susceptibility, and will likely not be equivalent across different life stages (see also [42]). They also point to opportunities to incorporate more complexity into our models of disease outbreaks in the laboratory and field, with the costs of sex-specific frailty on pathogen transmission (see discussion in [10]), as well as decomposing per-propagule infectivity (β) into sex-specific contact rates and per-propagule infectivity (*sensu* [61,85]) being obvious extensions. By bringing this expanded scope to studies of sex differences across a wider variety of taxa, we may arrive at a much better understanding of the spread of infectious diseases in nature—where sex ratios, age structure and pathogen density vary enormously.

## Data Availability

Data are available on figshare at https://doi.org/10.26180/22818179 [86]. Supplementary material is available online [87].
